# Efficacy of Pazopanib in the Treatment of Metastatic Malignant Giant Cell Tumor of Soft Tissue: A Case Report

**DOI:** 10.3390/curroncol29020064

**Published:** 2022-01-31

**Authors:** Tadashi Iwai, Naoto Oebisu, Manabu Hoshi, Naoki Takada, Hiroaki Nakamura

**Affiliations:** Department of Orthopedic Surgery, Osaka City University Graduate School of Medicine, 1-4-3 Asahi-Machi, Abeno-Ku, Osaka 545-8585, Japan; evis@med.osaka-cu.ac.jp (N.O.); hoshi@med.osaka-cu.ac.jp (M.H.); m2026957@med.osaka-cu.ac.jp (N.T.); hnakamura@med.osaka-cu.ac.jp (H.N.)

**Keywords:** giant cell tumor, soft tissue, recurrence, metastasis, pazopanib

## Abstract

Giant cell tumor of soft tissue (GCT-ST), histologically resembling the GCT of the bone, is a rare tumor. The tumor has been categorized to have low malignancy. Few reports of local recurrence or distant metastasis and the use of chemotherapeutic agents for metastatic GCT-ST exist. Herein, we report the efficacy of pazopanib in a 78-year-old Japanese woman with GCT in the intrinsic back musculature with both post-operative local recurrence and lung metastasis. The patient visited the hospital with a three-month history of a palpable mass in the intrinsic back musculature. Following magnetic resonance imaging, the tumor predominantly exhibited slight hyperintensity on T2-weighted images and intense heterogeneous enhancement on contrast-enhanced T1-weighted images. A percutaneous needle biopsy was performed, and the pathological diagnosis was GCT-ST. The patient underwent surgery, and three months later she presented with not only local recurrence but also multiple lung metastases. The patient was immediately treated with pazopanib 400 mg once daily. One month after initiating treatment, a partial response in the pulmonary lesions was observed, and stable disease (SD) effects lasted for 11 months without severe adverse effects. Therefore, pazopanib treatment for metastatic malignant giant cell tumor of soft tissue achieved reasonable success.

## 1. Introduction

Giant cell tumor of soft tissue (GCT-ST) is a rare cancer that was first described by Salm and Sissons in 1972 as a distinct entity [1]. GCT-ST resembles a giant cell tumor (GCT) of the bone, which has low malignancy [2,3]. This tumor, which has been described in numerous anatomical sites, including the upper and lower extremities (70% of tumors), trunk (20%), head and neck (7%), and tendon sheaths, can occur in both superficial and deep soft tissues [2,4]. The tumor exhibits biologically benign behavior [5]; therefore, removal of the mass with clear surgical margins almost guarantees no recurrence [6]. However, there is a report about an extremely rare GCT-ST with distant metastasis after surgical management [7]. Herein, we describe another rare case of GCT of the intrinsic back musculature with post-operative local recurrence and lung metastasis identified following a computer tomography (CT) scan and magnetic resonance imaging (MRI).

Furthermore, there are no previous reports about the effect of pazopanib treatment in elderly patients with metastatic GCT-ST. To the best of our knowledge, this is the first case report to present the efficacy of pazopanib in a patient with metastatic GCT-ST.

## 2. Case Presentation

A 78-year-old Japanese woman visited our department complaining of an enlarged mass localized in the intrinsic back musculature, which had been present for approximately three months. There was no tenderness or fever. Furthermore, no weight loss was noted during the initial visit, and her family history was unremarkable.

**Figure 1:**
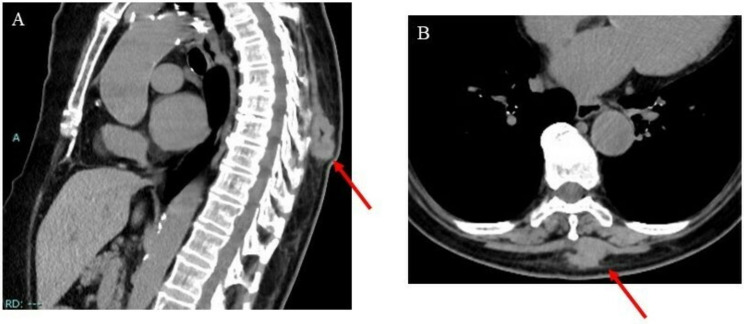
Images of the intrinsic back musculature of a 78-year-old female patient: (**A**) sagittal computed tomography (CT) image; (**B**) axial CT image.

A local CT scan revealed a 32 cm × 24 cm × 41 cm lesion superficially located in the intrinsic back musculature (Figure 1).

**Figure 2:**
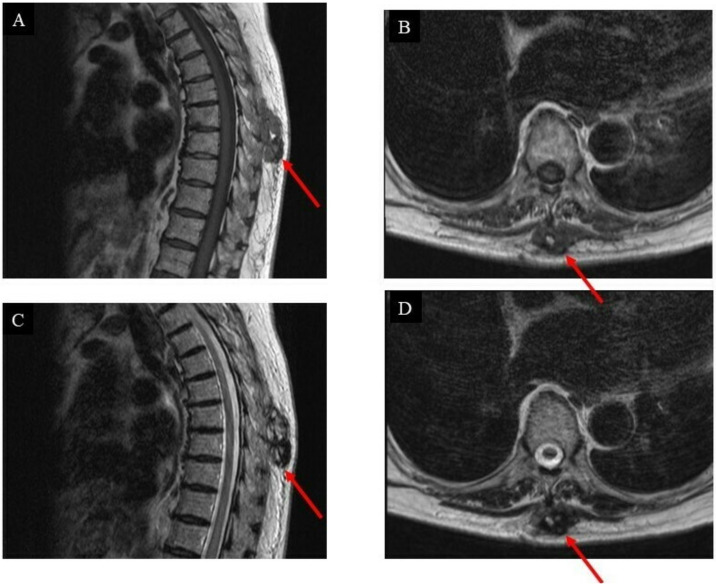
Images of the intrinsic back musculature of a 78-year-old female patient before treatment: (**A**) sagittal magnetic resonance imaging (MRI) T1-weighted sequence; (**B**) axial MRI T1-weighted sequence; (**C**) sagittal MRI T2-weighted sequence; (**D**) axial MRI T2-weighted sequence.

Moreover, on T2-weighted MRI images, the tumor appeared as a heterogeneous mass, whose main component was solid, mixed with a small portion of varied-sized cysts (Figure 2).

**Figure 3:**
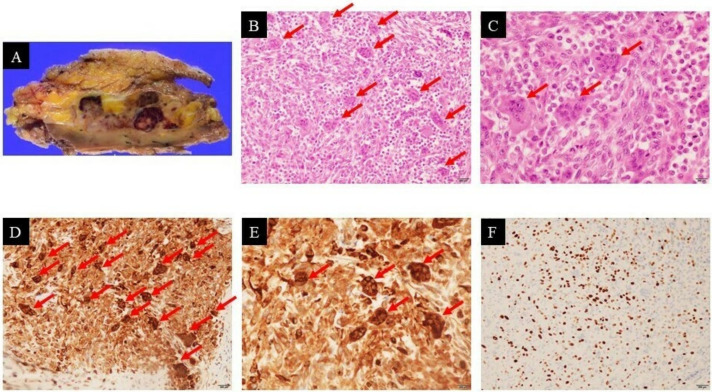
Pathological examination of the specimen confirmed giant cell tumor of soft tissue: resected specimen (**A**); hematoxylin–eosin staining (×200 (**B**), ×400 (**C**): The tumor was composed mainly of oval and spindle mononuclear cells (tumoral cells) and multinucleated giant cells (arrows)); immunohistochemical staining of CD68 showed cytoplasmic positivity (×200 (**D**), ×400 (**E**): Oval and spindle mononuclear cells (tumoral cells) and multinucleated giant cells (arrows)); Ki-67 labeling index was high (×200 (**F**)).

Following these observations, a core needle biopsy was performed, and the pathological diagnosis indicated a low-grade sarcoma. Based on this conclusion, wide resection of the tumor was performed. The resected specimen was approximately 45 mm × 25 mm × 40 mm in size, highly vascular, focally hemorrhagic, and adherent to fat tissue (Figure 3A).

After careful separation, the tumor was macroscopically excised completely; however, histological examination revealed incomplete resection (R1 resection), many blood-filled cavities similar to those present in aneurysmal bone cysts, a mixture of mononuclear cells and multinucleated osteoclast-like giant cells with high mitotic activity (38 figures per 10 HPFs), and no notable nuclear atypia (Figure 3B,C). Based on immunohistochemistry, the tumor showed a strong positive reaction in the giant cells to the histiocytic marker CD68 (Figure 3D,E). The Ki-67 labeling index ranged from 25% to 30%, indicating nuclear positivity (Figure 3F). The final pathological diagnosis was GCT-ST with low malignant potential. Some systematic evaluation (including lung CT scan) revealed no evidence of any other primary lesion or distant metastasis. Based on the American Joint Committee on Cancer (AJCC) staging system for soft tissue sarcoma (STS) of the trunk and extremities [8], the primary stage of the tumor was determined to be stage IA (G1T1NXM0).

Based on R1 resection, the additional wide resection was considered as the standard treatment. However, based on the strategy of demarcating surgical margins for bone and soft tissue sarcoma, it could be predicted that a large defect of the back would occur upon the removal of the tissues including vertebrae and ribs [9]. It would also have been very difficult to reconstruct complex posterior trunk defects [10]. Therefore, the procedure was not performed, considering the severity of the invasiveness.

**Figure 4:**
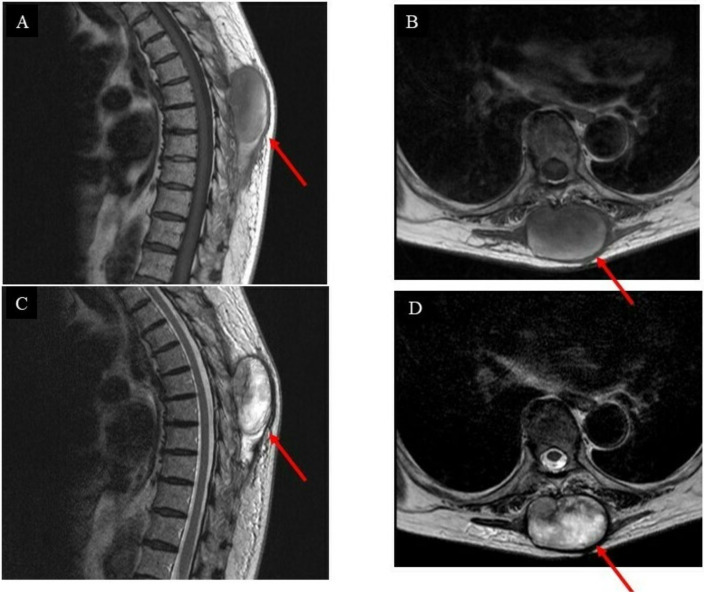
Images of the intrinsic back musculature of the patient three months after wide resection: (**A**) sagittal magnetic resonance imaging (MRI) T1-weighted sequence; (**B**) axial MRI T1-weighted sequence; (**C**) sagittal MRI T2-weighted sequence; (**D**) axial MRI T2-weighted sequence.

The first follow-up was three months after the surgery, and an MRI scan (Figure 4) was performed subsequently.

**Figure 5:**
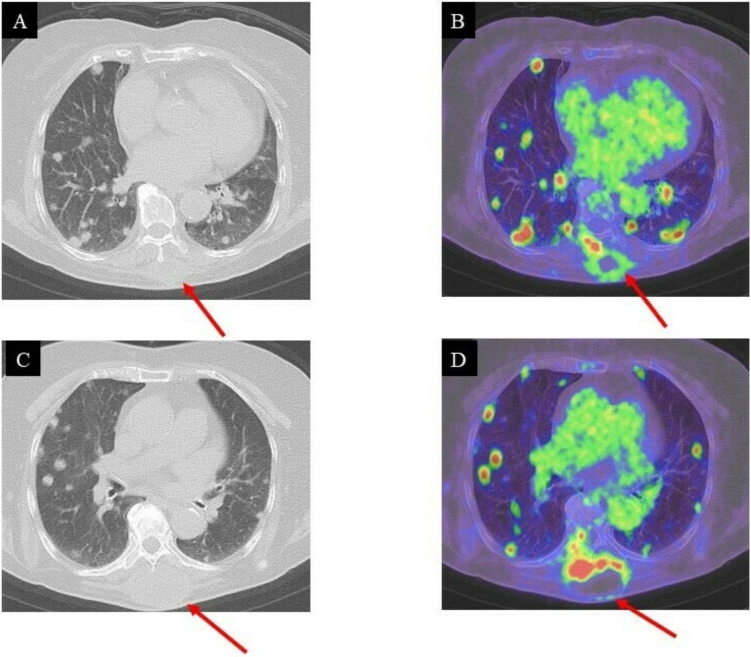
CT and PET/CT images of the lung three months after surgical resection: local recurrence and multiple pulmonary metastases; (**A**,**B**), (**C**,**D**): high FDG uptake in the intrinsic back musculature (SUV max = 14.96) and bilateral lung lesions (SUV max = 7.15–16.22), respectively.

Unfortunately, the tumor had recurred and was located at the surgical site. The pathological diagnosis indicated a low-grade GCT-ST after a second needle biopsy, and positron emission tomography–computed tomography (PET-CT) was conducted to investigate the metastasis (Figure 5A–D).

**Figure 6:**
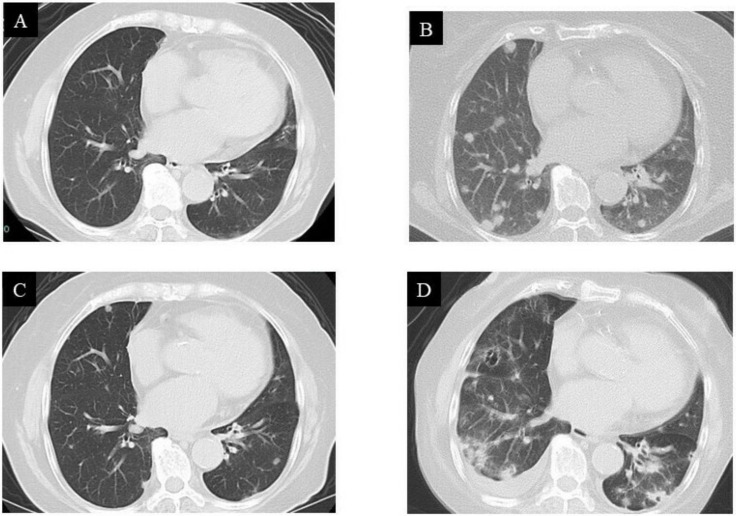
Computed tomography (CT) images of the lung: (**A**) no pulmonary metastases observed before treatment; (**B**) multiple pulmonary metastases observed three months after surgical resection; (**C**) metastases rapidly regressed after pazopanib treatment; (**D**) metastases recurred 11 months after pazopanib treatment.

While the lung CT images showed no metastatic lesions before the operation (Figure 6A), multiple diffuse pulmonary metastases were observed three months after surgical resection (Figure 6B). The patient was a 78-year-old female. She was advised to undergo chemotherapy, where doxorubicin would be administered via an intravenous (IV) injection, while pazopanib could be orally administered. Therefore, a pazopanib (Votrient^®^) regimen of 400 mg, once daily, was initiated to treat the metastatic GCT-ST. Regression of lung metastases was observed during the first month of treatment with pazopanib. Based on the Response Evaluation Criteria in Solid Tumors (RECIST) principles, treatment efficacy was evaluated as a partial response (Figure 6C). Nevertheless, an unfortunate rapid development of metastatic pulmonary lesions was observed after 11 months (Figure 6D), and the patient eventually succumbed to the disease. The patient and her family were duly informed about the data to be submitted for publication, and they provided their consent for this.

## 3. Discussion

Regarding pathological diagnosis, round or elliptical tumor cells were diffusely distributed in the tumor tissue (Figure 3B,C). These cells were more regular in shape and lacked obvious pathological mitotic figures, where a large number of osteoclasts were visible. These features of GCT-ST were very similar to those of undifferentiated pleomorphic sarcoma with giant cells, making it difficult to differentiate between the two tumor types. However, Folpe et al. had reported cases with the histopathologic features of undifferentiated pleomorphic sarcoma with giant cells lacking marked cytological atypia and reclassified them as “giant cell tumor of low malignant potential” [11]. Furthermore, many undifferentiated pleomorphic sarcomas showed focal immunoreactivity for smooth muscle actin [12]; however, in this case, the resected specimen was negative for smooth muscle actin. Therefore, the final pathological diagnosis was GCT-ST with low malignant potential.

GCT-ST occurs in both superficial (70%) and deep (30%) soft tissues [2,3]. Surgical excision, the most accepted management technique, has been performed in almost all reported cases, leading to a low recurrence rate (approximately 12%) [2,7]. Furthermore, lung metastasis was reported to be much lower than local recurrence [2,7]; therefore, few reports have discussed the treatment strategy of metastatic GCT-ST. In this report, we provide important information regarding surgery, post-operative chemotherapy, and oncological outcomes of a patient with metastatic GCT-ST.

Positron emission tomography (PET)/CT with Fluorodeoxyglucose (FDG) is a crucial non-invasive diagnostic tool for the management of patients with soft tissue sarcomas [13]. Previous reports suggested that FDG avidity was related to strong hexokinase II activity in giant cells [14]. Giant cells within a lesion are the main cause of increased FDG avidity, which causes false-positive diagnoses of malignancy through PET-CT [14]. Nevertheless, PET-CT has been widely used to differentiate benign from malignant tumors as well as to detect distant metastasis [15,16]. FDG uptake in GCT was reported to be of varied values (SUV max = 3–25) [17,18]. In the present case, the FDG uptake in the intrinsic back musculature (SUV max = 14.96) and bilateral lung lesions (SUV max = 7.15–16.22) was high three months after surgical resection. Therefore, PET-CT for GCT-ST was considered to be suitable for detecting distant metastases.

Pazopanib (Votrient^®^), a tyrosine kinase inhibitor that targets the vascular endothelial growth factor receptor, is a second-line treatment option for advanced and metastatic STS after treatment with anthracyclines fails [19,20]. Recently, Grünwald et al. indicated that pazopanib was non-inferior to anthracyclines, making it a promising therapeutic option in the first-line treatment of advanced and metastatic STS in patients aged 60 years or older [21]. Moreover, in Japan, the currently approved dose of pazopanib (800 mg) has long been controversial, owing to its adverse effects [22]. Therefore, it was re-examined to verify the ideal dose for Japanese by Tanaka et al. [22] A dose of 400 mg was reported to be effective and well-tolerated by more than half of the treated patients [22]. Therefore, in the present case, we decided to initiate the patient on 400 mg of pazopanib daily. Tanaka et al. also suggested that therapeutic drug monitoring of pazopanib was necessary for adequate dose adjustment [22]. Tanaka et al. had shown that 35% of patients who received 400 mg pazopanib had an ineffective plasma concentration of the drug [22]. Therefore, drug monitoring of pazopanib might have been necessary during pazopanib therapy to maintain the best effect. However, we did not perform the monitoring. Nevertheless, the human serum albumin (Alb) levels of the patient were maintained at about 3.9 mg/mL. This is noteworthy because Tanaka et al. had also indicated that the albumin level was significantly associated with effective pazopanib concentrations. Moreover, the patient had no history of severe medical conditions, despite being 78 years of age. Therefore, a dose of 400 mg was being administered daily, wherein the effective pazopanib concentrations were maintained in the patient.

Regression of lung metastases was observed during the first month of treatment with pazopanib. Based on the RECIST criteria, treatment efficacy was evaluated as a partial response (Figure 6C). Verschoor and Gelderblom reported that approximately 15% of patients with lung metastasis from STS who received pazopanib developed pneumothorax [23]. However, pneumothorax was not observed after pazopanib administration in the present case. Although thrombocytopenia has also been reported to be another main adverse event [24], it was not observed in this case.

The pazopanib for metastatic STS (PAzopanib expLorEd in sofT-Tisue Sarcoma—a phasE 3 study (PALETTE)) study reported a median overall survival (OS) of 11.2 months following treatment initiation (12.5 months in the pazopanib group). Nakamura et al. indicated that good performance status and being female were favorable prognostic factors for OS [24]. In the present case, the patient was female, and her OS was approximately 14 months, which is consistent with previous reports. Furthermore, a positive resection margin (R1/R2) is considered to be a poor prognostic factor for local recurrence of STS [25]. The median PFS for all patients of the previous report was approximately four months [24], while her progression-free survival (PFS) was about three months. This may be related to the R1 resection.

Radiotherapy (RT) is an effective adjuvant therapy administered after surgery for adult STS to prevent local recurrence [26]. According to the National Comprehensive Cancer Network (NCCN) guidelines, RT should be performed to treat stage II or III STS [27]. In the case of stage I, when clear margins cannot be obtained, RT needs to be considered [26]. However, for stage IA tumors (<5 cm), a wait-and-see attitude can be adopted [26]. Therefore, based on the clinical stage IA of the present case, post-operative RT was not administered even after R1 resection.

## 4. Conclusions

We report the case of a patient who presented with primary malignant GCT-ST located in the intrinsic back musculature with post-operative local recurrence and lung metastases. Post-operatively, a close follow-up is essential because of the malignant potential of the tumor, leading to not only local recurrence but also distant metastases. In the present case, pazopanib treatment was effective, as previously reported, and its efficacy lasted for 11 months. However, detailed data regarding the efficacy of pazopanib in the treatment of metastatic malignant GCT-ST are scarce, thus marking the significance of publishing such data. Our decision to choose pazopanib as first-line treatment for this patient may be controversial because the study by Grünwald et al. did not include patients with metastatic malignant GCT-ST. In general, evidence for this rare entity is very limited. Therefore, further studies are required to conclusively verify the efficacy of pazopanib in patients with metastatic malignant GCT-ST.

## Data Availability

All the data are available from the corresponding author upon reasonable request.
